# Mst1/2 signalling to Yap: gatekeeper for liver size and tumour development

**DOI:** 10.1038/sj.bjc.6606011

**Published:** 2010-11-23

**Authors:** J Avruch, D Zhou, J Fitamant, N Bardeesy

**Affiliations:** 1 Department of Molecular Biology, Massachusetts General Hospital, Simches Research Building, 6408, 185 Cambridge Street, Boston, MA 02115, USA; 2 Diabetes unit, Massachusetts General Hospital, Simches Research Building, 6408, 185 Cambridge Street, Boston, MA 02115, USA; 3 Cancer Center, Massachusetts General Hospital, Simches Research Building, 4216, 185 Cambridge Street, Boston, MA 02115, USA; 4 Department of Medicine, Harvard Medical School, Boston, 185 Cambridge Street, CPZN4216, MA 02115, USA

**Keywords:** liver cancer, hepatocellular carcinoma, cholangiocarcinoma, oval cells, Hippo, Rassf polypeptides, tumour suppressor pathway

## Abstract

The mechanisms controlling mammalian organ size have long been a source of fascination for biologists. These controls are needed to both ensure the integrity of the body plan and to restrict inappropriate proliferation that could lead to cancer. Regulation of liver size is of particular interest inasmuch as this organ maintains the capacity for regeneration throughout life, and is able to regain precisely its original mass after partial surgical resection. Recent studies using genetically engineered mouse strains have shed new light on this problem; the Hippo signalling pathway, first elucidated as a regulator of organ size in *Drosophila*, has been identified as dominant determinant of liver growth. Defects in this pathway in mouse liver lead to sustained liver overgrowth and the eventual development of both major types of liver cancer, hepatocellular carcinoma and cholangiocarcinoma. In this review, we discuss the role of Hippo signalling in liver biology and the contribution of this pathway to liver cancer in humans.

Liver cancer is the fifth most common cancer worldwide and the third leading cause of cancer death (Farazi and DePinho, 2006). The two most common primary liver cancers, hepatocellular carcinoma (HCC) and cholangiocarcinoma (CC) exhibit distinct histological and molecular profiles. The conventional view has been that HCC and CC arise from hepatocytes and bile duct cells (cholangiocytes), respectively, however the existence of a subset of liver tumours showing mixed HCC/CC histology has suggested an origin from a bipotential progenitor cell (Zhang *et al*, 2008). In terms of risk factors, a common shared theme is the role of chronic tissue damage, viral hepatitis and inflammation, suggesting an important role for repeated cycles of cell injury, death and regeneration in disease predisposition. Both of these tumour types carry a poor prognosis with potentially curative surgery only possible in the subset of patients diagnosed with early disease. Some responses are seen with conventional and targeted chemotherapies, however, the impact on overall survival is modest (Villanueva *et al*, 2010). Therefore, the elucidation of the molecular pathogenesis of HCC and CC is needed to improve therapeutic approaches for these diseases.

A series of recent studies have demonstrated that the *Mst1*, *Mst2*, *Sav1* (also known as *WW45*), and *Yap* genes are important for growth control and tumourigenesis in the liver. Notably, each of these genes is orthologous to a member of the *Drosophila* Hippo tumour suppressor pathway, a gene network that seems to monitor cell–cell contact and cell polarity, and thereby restrict organ overgrowth (reviewed in Reddy and Irvine, 2008). Correspondingly, the pathway in mammals is revealed as an evolutionarily conserved mechanism for both organ size control and tumour suppression. Not surprisingly however, the expansion of these genes in mammals is also accompanied by a tissue-specific diversification of their functions and of the architecture and regulation of the pathway. In this study, we review distinct aspects of Hippo signalling operative in the liver, discuss the impact of this pathway on proliferative control in different hepatic cell lineages, and evaluate the relevance of defective Hippo signalling to human liver cancer.

## Mst1/2 kinases and the Hippo pathway

The Mst1/2 kinases are cytosolic Ste20-related kinases activated by autophosphorylation (Creasy *et al*, 1996). Designated class II GC kinases, Mst1 and Mst2, share 76% sequence identity and contain an N-terminal catalytic domain, followed successively by an autoinhibitory segment and a coiled-coil SARAH domain that mediates hetero- and homo-dimerisation. The SARAH domain is present in a small number of additional mammalian proteins, including Sav1 and the Rassf/Nore family (Hwang *et al*, 2007; Avruch *et al*, 2009), each of which are non-catalytic adaptors that have been functionally linked to Mst1/Mst2 (see below). Overexpression of either Mst1 or Mst2 induces apoptosis in many transformed cell lines and are themselves activated under a variety of apoptotic conditions (Graves *et al*, 1998).

**Figure 1:**
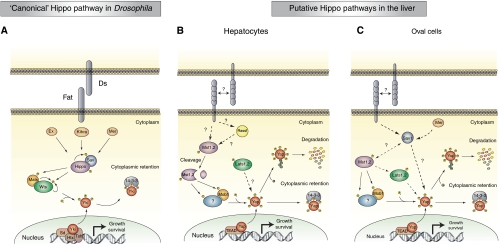
Hippo pathway circuitry in *Drosophila* and in the mammalian liver. (**A**) Model of *Drosophila* Hippo signaling. Signaling may be initiated in response to the atypical cadherin Fat receptor activation through Ds binding. Signals are transduced through the FERM domain-containing cytoskeleton-associated protein, Merlin (Mer) and Expanded (Ex), and by Kibra, a protein that interacts with Mer and Ex. The Hippo kinase interacts with and phosphorylates the scaffold protein Sav promoting Hippo-mediated phosphorylation of the adaptor Mats and the Wts kinase. Wts is thereby activated and phosphorylates the transcriptional coactivator Yki. Phosphorylation of Yki induces its cytoplasmic retention through 14-3-3 binding. In the absence of Hippo pathway activation, Yki is mainly located in the nucleus, wherein it binds and activates various DNA biding transcription factors including Sd, Htx and Tsh to induce expression of genes implicated in cell growth and survival. **B** and **C** components of the Hippo pathway are highly conserved in mammals, wherein they have a critical role in proliferative control in the liver (mammalian orthologues are indicated with the same colour scheme as the corresponding *Drosophila* proteins). Although the circuitry is incompletely defined, it seems that two distinct models either in the oval cells or in hepatocytes can be proposed based on recent studies. In both cell types, inhibition of the Yki orthologue, Yap, is thought to be a critical output of the pathway. Defects upstream of Yap result in nuclear retention of Yap, which functions in association with DNA-binding transcription factors, for example, the TEAD domain transcription factors (orthologues of Sd) to regulate the expression of genes that control cell growth and survival. (**B**) In hepatocytes, Mst1/2 are required to phosphorylate Mob1. By analogy to *Drosophila*, phospho-Mob1 is likely to facilitate activation of an intermediary kinase, which phosphorylates Yap, resulting in both cytoplasmic retention by 14-3-3 binding, as well as cytoplasmic degradation after ubiquitinylation. Lats1/2 activity are unchanged by Mst1/2 inactivation suggesting the existence of a yet to be defined Mst1/2-regulated Yap kinase. The majority of the catalytically active Mst1/2 in the liver is in a truncated form that lacks the autoregulatory carboxy-terminus. The upstream activators of Mst1/2 are not defined, although Rassf family proteins, could link Mst1/2 to extracellular signals facilitating activation before the proteolytic cleavage. Sav1 does not seem to have a role in Yap regulation in hepatocytes. (**C**) In oval cells, Sav1 controls total Yap protein levels and levels in the nucleus through yet to be defined mechanisms. The relationship of Sav1 to the Mst1/2 activation state and the phosphorylation of Yap is not clear, although the Mst1/2-controlled phosphorylation of Yap-Ser127 is unaffected by Sav1 inactivation. Mst1/2 have not been studied specifically in oval cells, however, the increase in oval cells following Mst1/2 inactivation indicate an important regulatory role for these kinases in oval cells. The components upstream of these pathways are incompletely defined, although cell–cell contact is likely to be an important stimulus.

The Hippo pathway emerged from *Drosophila* screens for genes whose loss results in organ overgrowth. The first element identified was the Lats kinase, (Xu *et al*, 1995) and evidence for a pathway was provided by the elucidation of Salvador (Tapon *et al*, 2002), which encodes a non-catalytic protein that binds directly to Lats and has a similar LOF phenotype. The Mst1/Mst2 orthologous kinase Hippo, identified the following year as a growth suppressor, was shown to bind Salvador and phosphorylate Lats (reviewed recently by Reddy and Irvine, 2008). Overgrowth in this pathway reflects both increased proliferation and a failure of developmental apoptosis. Several other genes giving phenotypes similar to Hippo were identified and found to interact genetically and biochemically. The core of the pathway involves a kinase cascade in which Hippo phosphorylates the Lats/Warts kinase, which in turn multiply phosphorylates Yorkie/Yap, a transcriptional co-activator that controls several classes of transcription factors, including Scalloped (TEAD domain proteins), homothorax and SMADs (Oh and Irvine, 2010; Figure 1A). The scaffold protein Salvador/shar-pei (the Sav1 orthologue), which binds both Hippo and Lats/Warts, links the pathway to upstream signals. The noncatalytic protein MATS (MOBKL1A/B) is a Hippo substrate that once phosphorylated, binds to and promotes Lats autophosphorylation and activation (Wei *et al*, 2007; Praskova *et al*, 2008). Genetic studies show that elimination of Yorkie reverses the loss-of-function phenotype of all the other genes, indicating that the primary function of the pathway is the inhibition of Yorkie/Yap (Huang *et al*, 2005). One crucial mechanism of Yorkie inhibition is through Lats/Warts-catalysed phosphorylation, followed by 14-3-3 binding and nuclear exit (Huang *et al*, 2005; Ren *et al*, 2010). The pathway components and their arrangement upstream of Hippo is less well defined; among the positive regulators are submembrane actin-associated FERM domain proteins, such as Merlin, whose human orthologue is the NF2 tumour suppressor, and an atypical cadherin called Fat (reviewed in Reddy and Irvine, 2008). Thus, although the wiring between the cell surface and Hippo is unclear, cell–cell contact is likely to be an important activating stimulus (Zhao *et al*, 2007).

In addition to Mst1/2, Sav1 and Yap, the other core components of the ‘Hippo pathway’ are conserved in mammals (Warts=Lats1 and 2; MATS=Mobkl1a and b) and each can rescue the *Drosophila* LOF phenotypes. Initial studies in mammalian cell culture and *in vitro* indicated that the regulation of the pathway in mammals is comparable with that seen in the fly; overexpression of combinations of Mst1/2, Lats1/2 and Sav1 results in Yap phosphorylation (at Ser127) and nuclear exit, whereas depletion of Lats1/2 in some cancer cell lines inhibits Yap phosphorylation. *In vitro*, Mst2 can phosphorylate purified Lats1/2, and the latter directly phosphorylates Yap (Ser127) (reviewed in Oh and Irvine, 2010). As regards upstream regulation, mouse embryonic fibroblasts (MEFs) on reaching confluence exhibit increased Yap(Ser127) phosphorylation and reduced nuclear abundance, suggesting that cell–cell contact activates the pathway (Zhao *et al*, 2007). The importance of Yap phosphorylation and nuclear exit to contact-induced arrest of proliferation is shown by the ability of a non-phosphorylatable Yap mutant to bypass contact inhibition. Notably, MEFs deficient in either NF2/Merlin (Curto and McClatchey, 2008) or Lats2 (McPherson *et al*, 2004) also lack normal contact inhibition. Although these observations suggested the operation in mammalian cells of a pathway identical to that in *Drosophila*, recent studies demonstrate that the pathway upstream of Yap as well as the regulation and functions of Mst1 and 2 are each more diverse and tissue specific than anticipated from genetic analysis of the developing *Drosophila* eye (see below).

## Overview of growth control in the liver

**Figure 2:**
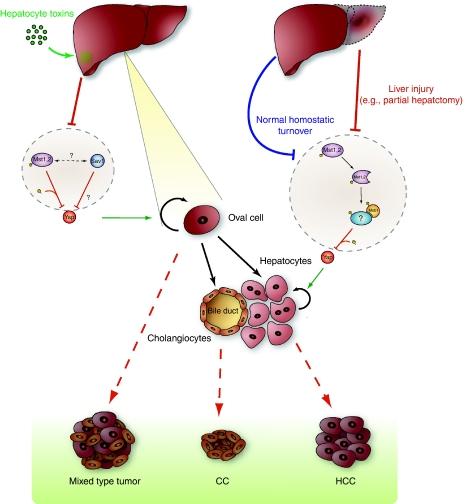
Model for the role of the Hippo pathway during liver regeneration and tumorigenesis. The normal adult liver is mainly composed of two parenchymal cell types, the hepatocytes and the cholangioctyes that surround the bile ducts. During embryogenesis (not shown), a common progenitor cell gives rise to these both of these cell types. In the adult, the liver is largely quiescent. The gradual replacement of cells during normal physiologic turnover is accomplished by the proliferation of the differentiated liver cells. Similarly, in response to various forms of liver injury or to partial hepatectomy, the liver mass is restored through cell cycle entry of remaining parenchymal cells. In contrast, when parenchymal cells are unable to proliferate (e.g., in response to hepatocyte toxins), rare cells associated with the bile ducts known as oval cells expand and then differentiate to restore liver mass. The Mst1/2 kinases seem to control hepatocyte quiescence by the inhibition of Yap activity. This inhibition may be periodically relieved during normal homostatic turnover, as well as in response partial hepatectomy. The quiescence of oval cells seem to be controlled both by Mst1/2 and Sav1, and again, Yap is a candidate downstream target of the pathway. Sustained defects in Mst1/2 result in hepatocyte and oval cell proliferation and the development of HCC and tumors of mixed HCC and CC histology. Sav1 defects produce comparable phenotypes except that no hepatocyte proliferation is observed.

Growth control in the liver has a number of unusual features compared with that in other organs. Adult liver cells are largely quiescent, dividing approximately once/year; nevertheless, differentiated adult hepatocytes, rather than multipotent stem cells, are the source for tissue replenishment of cell turnover in the undamaged liver (Ponder, 1996). The liver is also characterised by a remarkable regenerative capacity (reviewed in Michalopoulos, 2007). In response to removal of up to 70% of liver tissue, liver mass is restored through cell cycle entry of remaining adult hepatocytes and cholangiocytes (bile duct cells; Figure 2, right hand side). If however hepatocyte proliferation is suppressed (e.g., in response to hepatotoxins), facultative liver stem cells (oval cells), a very minor compartment in the normal liver, expand and differentiate into both hepatocytes and cholangiocytes, sufficient to restore liver volume (Figure 2, left hand side). The transcriptional programme of post-hepatectomy hepatocyte proliferation differs strongly from that of injury-related, oval cell-mediated regeneration (Otu *et al*, 2007), the latter resembling more closely that of proliferating fetal hepatoblasts (Bird *et al*, 2008; Si-Tayeb *et al*, 2010).

Although numerous models of HCC have been described, no master regulators of either hepatocyte or oval cell quiescence had been identified until recently. As regards oval cells, selective expansion of these cells is observed in mice that overexpress TWEAK (Jakubowski *et al*, 2005), a ligand for Fn14, a member of the TNF receptor family. Moreover, antibody-mediated blockade of this pathway suppresses oval cell expansion in a liver injury model. Hence, Tweak signalling can overcome oval cell quiescence and is required for sustained proliferation of these cells. Nevertheless, TWEAK and Fn14 seems to be constitutively expressed (Burkly *et al*, 2007), so that additional mechanisms must restrain oval cell expansion as well as maintain hepatocyte quiescence.

## The Mst1/2-Yap1 module in the regulation of quiescence and proliferation in liver

The first indication that Yap is important in proliferative control in the liver came from studies of p53 null primary fetal liver cells (hepatoblasts) engineered to overexpress the c-Myc oncogene (Zender *et al*, 2006). When implanted into mouse liver, these cells developed into HCC's that showed recurrent amplification of the chromosomal locus harbouring Yap. Knockdown of Yap in HCC-derived cell lines attenuated tumourigenicity, whereas combined Yap and c-Myc overexpression accelerated HCC development, providing direct evidence that Yap functions as an oncogene in the liver. Extension and striking confirmation of this conclusion was provided by the generation of transgenic mice engineered to overexpress Yap under a doxycycline-inducible promoter (Camargo *et al*, 2007; Dong *et al*, 2007). In this study, Yap expression was induced in adult mice either ubiquitously or specifically in the liver, in both cases leading to immediate and pronounced liver overgrowth, associated with marked hepatocyte proliferation and a resistance to apoptosis induced by administration of anti-FAS antibody. Although liver mass increased five-fold, removal of doxycycline after 8 weeks resulted in a reversion to normal size and architecture within 2 weeks. If, however, expression of the Yap transgene was sustained, multifocal HCC developed within several months (Dong *et al*, 2007; Table 1). Hence, Yap expression overrides hepatocyte quiescence and overall liver size control, and (as with *Drosophila* Yorkie) desensitises liver cells to apoptosis. Yap overexpression also led to aberrant proliferation in the intestinal epithelium, pancreas and skin, although neither gross organ enlargement or tumours were reported in these organs (Camargo *et al*, 2007).

Recent studies by our group and others using mouse knockouts of the Mst1/Mst2 kinases (Zhou *et al*, 2009; Lu *et al*, 2010; Song *et al*, 2010) and the Sav1 scaffold (Le *et al*, 2010; Lu *et al*, 2010) have uncovered critical roles for these putative upstream elements of the Hippo pathway in proliferation control in the liver (Table 1). Mst1 and Mst2 single knockout mice are viable and do not exhibit organ overgrowth or tumour development. Mst1^−/−^Mst2^−/−^ double-knockout mice exhibit early embryonic lethality (Oh *et al*, 2009; Zhou *et al*, 2009), whereas both Mst1^−/−^Mst2^+/−^ and Mst1^+/−^Mst2^−/−^ mice are viable and fertile. Notably, however, the mice born with only a single allele of Mst1 or Mst2 develop spontaneous liver tumours associated with loss of the remaining wild-type Mst1 or Mst2 allele in the tumours (Zhou *et al*, 2009). The spontaneously arising tumours were all HCCs, although examination of a large cohort of mice revealed that ∼10% had elements of mixed HCC/CC histopathology. At euthanisation, tumours were not observed in other organs of these mice. These results establish that Mst1 and Mst2, in a redundant manner, function as potent tumour suppressors in liver.

The use of the conditional alleles and the albumin (Alb)–Cre strain enabled study of liver specific homozygous Mst1/Mst2 inactivation (Zhou *et al*, 2009; Lu *et al*, 2010; Song *et al*, 2010). Compound Alb–Cre, Mst1/Mst2 mutant mice show marked liver enlargement at 4–5 weeks age and require euthanisation by 4–5 months because of multiple large liver tumours. The precancerous livers of these mice exhibit overproliferation of hepatocytes as well as a marked expansion of the oval cell compartment. The liver tumours show features of both HCC and mixed HCC/CC, with HCC comprising the larger proportion of the tumour area. Inasmuch as oval cells are bipotential liver progenitors, the frequent occurrence of mixed tumour histopathology is probably attributable to the malignant transformation of these cells resulting in tumour cells with dual differentiation programs.

Acute Mst1/Mst2 deletion in the adult liver, achieved either by intravenous injection of adenovirus expressing Cre recombinase (Zhou *et al*, 2009), or by tamoxifen induction of MMTV–CreERT or CAAGS–CreERT (Lu *et al*, 2010; Song *et al*, 2010) produced an intermediate tumour phenotype consisting of both HCC and mixed HCC/CC histology. Remarkably, acute Mst1/Mst2 deletion caused a doubling in liver mass associated with marked proliferation of hepatocytes, a substantial expansion of oval cell, resistance to FAS-induced apoptosis, and rapid development of liver cancer (Zhou *et al*, 2009; Lu *et al*, 2010). Comparing the various models, it seems that the timing and/or mechanism of Mst1/Mst2 inactivation influences the ensuing phenotype; deletion early in life in the Alb–Cre model is associated with the most marked oval cell expansion and the highest proportion of mixed HCC/CC histology in the liver tumours, whereas the tumours arising spontaneously in Mst1^−/−^/Mst2^+/−^ mice, which have little or no oval cell expansion before the random allele loss, are overwhelmingly HCC. In *Drosophila*, elimination of Yorkie is alone sufficient to revert the Hippo and Salvador (Sav1) loss-of-function phenotypes. Consistent with this, acute inactivation of Mst1/Mst2 in the liver is associated with rapid loss of Yap(Ser127) phosphorylation, increased Yap nuclear localisation and polypeptide abundance (Zhou *et al*, 2009). Yap is regulated by the ubiquitin–proteosome machinery via interaction with the E3-ligase *β*-TRCP, and it seems that Yap(Ser381) phosphorylation promotes Yap degradation (Zhao *et al*, 2010); the loss of this phosphorylation likely contributes to the rise in Yap protein levels in the Mst1/Mst2 KO liver. The changes in Yap phosphorylation, localisation and abundance seen after acute Mst1/2 inactivation are further accentuated in the subsequent Mst1/2 null HCC's and in cell lines derived therefrom. Yap knockdown in these Mst1/2-deficient HCC cell lines leads to massive cell death and cell cycle arrest; similarly, restoration of Mst1 expression in these cells restores Yap (Ser127) phosphorylation and engenders cell cycle arrest and apoptosis. Hence, the Mst1/Mst2-dependent inactivation of Yap is a critical tumour suppressor mechanism.

Two groups have described liver specific inactivation of Sav1/WW45 (Le *et al*, 2010; Lu *et al*, 2010; Table 1). The Alb–Cre-mediated excision of Sav1 results in a modest enlargement of the liver, plateauing at ∼1.5-fold over wild type, as compared with the four- to five-fold enlargement seen with Mst1/Mst2 double knockout. The Sav1-deficient livers exhibit a marked expansion of oval cells, however in contrast to the Mst1/Mst2-deficient livers, the Sav1 null livers do not show a parallel overproliferation of adult hepatocytes (Le *et al*, 2010). Liver tumours arose in Sav1^+/−^ mice and more reliably in the Alb–Cre Sav1 lox/lox mice and CAGGS–CreERT Sav1 lox/lox mice; nearly all of these were of a mixed HCC/CC histology. Moreover, these tumours were usually seen after 12 months age, considerably later than in the Mst1/Mst2 null livers. The liver of Alb–cre Sav1 null mice show a progressive increase in Yap polypeptide greater than that seen in Mst1/Mst2 null livers, with highest levels present in the Sav1 null liver tumours. Although Mst1 and Lats1 abundance also increase and Yap(Ser127) phosphorylation persists, the nuclear abundance of Yap is nevertheless also increased. The functional importance of Yap to the hyperproliferative behaviour of Sav1 null oval cells, although likely, remains to be demonstrated. It is notable that despite the evidence that Yap is activated in oval cells harbouring Hippo pathway defects, oval cell expansion in mice overexpressing Yap in the liver has not been described (Dong *et al*, 2007). This may reflect the relative activity in different cell lineages of the promoter used to drive Yap expression in these studies. In this regard, it is notable that the Yap transgenic mice show expansion of undifferentiated progenitor cells in the intestine and other tissues (Camargo *et al*, 2007). The changes in the Alb–cre Sav1 null livers seem to be attributable entirely to the oval cell compartment; adeno–Cre infection of isolated Sav1ff oval cells *in vitro* results in increased abundance and phosphorylation of Yap and Lats1 as well as more Mst1 polypeptide, paralleling the changes seen in the Alb–cre Sav1 null liver, whereas Sav1 deletion from isolated hepatocytes gives little change in the abundance or phosphorylation of these elements (Le *et al*, 2010). Overall the data seem to point to a general role for Mst1/Mst2 in regulation of liver cell proliferation, and a restricted function of Sav1 in oval cells (Figures 1 and 2).

The co-occurrence of marked oval cell expansion and mixed HCCs/CCs in the Sav1-deficient mice suggests the tumours arise from transformed oval cells that retain some capacity for hepatocytic and cholangiocytic differentiation. The broader induction of cell proliferation associated with Mst1/Mst2 inactivation appears to indicate that multiple cell lineages may undergo transformation in this context.

## Unexpected and unexplained features of the Hippo pathway in liver

Surprisingly, in contrast to *Drosophila*, Zhou *et al* (2009) find that Lats1/2 do not appear to serve as the direct, Mst1/Mst2-activated Yap kinases in hepatocytes (Figure 2B). Whereas Yap phosphorylation is markedly reduced by the acute elimination of Mst1/2 with adenoviral–cre, they find that phosphorylation of Lats1/Lats2 in liver is not significantly changed, nor is it altered by restoration of Mst1 into Mst1/2 null HCC cells; in contrast Le *et al* (2010) find Lats1/2 phosphorylation to be diminished in the Alb–cre/Mst1/Mst2 double knockout liver. Chromatographic separation of liver extracts and assay for Yap(Ser127) kinase demonstrates two peaks of activity; acute inactivation of hepatic Mst1/2 selectively eliminates one peak, whose elution is entirely distinct from that of immunoreactive Lats1 and Lats2 (Zhou *et al*, 2009). These data point to the existence of an novel, as yet unidentified intermediary kinase downstream of Mst1/Mst2 that is critical for Yap(ser127) phosphorylation in the liver; identification and elimination of this putative novel Mst1/Mst2-regulated kinase will be required to establish its physiologic role as a pathway component and liver tumour suppressor.

The unanticipated finding that Sav1 inactivation in the liver selectively affects the oval cell population, with little or no impact on pathway components in hepatocytes (Le *et al*, 2010; Lu *et al*, 2010) indicates that the resultant oval cell proliferation is a cell intrinsic response rather than a response to a hepatocyte-generated damage signal; whether this is true of the oval cell proliferation in the Mst1/Mst2 null liver is unresolved.

The Alb–cre mediated inactivation of a floxxed Neurofibromatosis 2 (NF2) gene results in the massive expansion of a periportal, CK2 positive cell population, variously called oval cells (Benhamouche *et al*, 2010) or biliary epithelial ‘hamartomas’ (Zhang *et al*, 2010); this results in gross liver enlargement with the subsequent development of HCC, CC or tumours of mixed histology. The selective oval cell expansion of the NF2-deficient mouse liver is similar to, but more pronounced than that described for the Sav1-deficient liver but differs from the expansion of both hepatocytes and oval cells seen with Alb–cre-mediated Mst1/Mst2 double knockout. The NF2 null liver phenotype is effectively suppressed by treatment of the NF2-deficient mice with erlotinib (Benhamouche *et al*, 2010), an inhibitor of the EGFR (and other) kinases, or by heterozygous inactivation of Yap1 (Zhang *et al*, 2010). The Alb–cre-mediated biallelic inactivation of Yap1 in liver *per se* causes a failure of bile duct development and hepatocyte apoptosis, both *in vivo* and in *ex vivo* culture (Zhang *et al*, 2010). The suppressive effect of Yap1 deletion on the overgrowth and tumourigenesis of the NF2-deficient liver is specific, in that Yap1 deletion has no impact on tumourigenesis in KiRas mutant livers. The evidence implicating NF2 in the negative regulation of yorkie in *Drosophila* and Yap1 in cell culture is strong; whether NF2 downregulation of EGFR signalling is an independent, parallel output of NF2 in oval cells or involves Yap1 as an intermediate is not clear. Moreover NF2 deficiency in humans is not accompanied by hepatic tumourigenesis, and the role of either pathway in the tumours resulting from human NF2 deficiency, as compared to NF2 inhibition of the CRL4^DCAF1^ ubiquitin ligase (Li *et al*, 2010) is an open question.

## Mechanisms of Mst1/Mst2 regulation in the liver

Three mechanisms for the physiologic activation of endogenous Mst1/2 are described thusfar, each involving phosphorylation within the activation loop (Praskova *et al*, 2004). In the canonical Hippo pathway, Sav1 is needed not only to facilitate Lats1/2 activation by Mst1/2, but also to enable Mst1/2 activation. Thus in Sav1 null keratinocytes, the ability of extracellular Ca^++^ to activate Mst1/2 activation loop phosphorylation is markedly impaired (Lee *et al*, 2008). The biochemical mechanism by which Sav1 mediates Mst1 activation is unclear; Mst1/2/Hippo associate with Sav1/salvador through their mutual SARAH domains, but this alone is not sufficient to cause activation.

A second mechanism for Mst1/2 activation is through their association with the Rassf family of polypeptides (Avruch *et al*, 2009). The major expressed isoforms of Rassf (1–6) all contain at their carboxyterminus a canonical ras–rap association (RA) domain followed by a SARAH domain. The RA domain enables binding to the GTP-charged forms of several Ras family GTPases, whereas Mst1/2 is capable of heterodimerisation with all such Rassf polypeptides through their mutual SARAH domains (Khokhlatchev *et al*, 2002). The affinity of the Rassf5/Nore1SARAH domain for the Mst1 SARAH domain greatly exceeds that of either homodimer (Hwang *et al*, 2007), and constitutive Rassf5/Mst1 and Rassf1/Mst1 heterodimers are evident in cells and tissues (Praskova *et al*, 2004). Although the Sav1 and Rassf5/Nore1 SARAH domains seems to interact with different surfaces on the Mst1 SARAH domain (Hwang *et al*, 2007), there is conflicting data as to whether endogenous heterotrimers occur (Polesello *et al*, 2006; Guo *et al*, 2007); therefore, whether Sav1 and Rassf mediate entirely independent pathways that each contain Mst1/2 or pathways that physically converge at Mst1/2 is currently unresolved. Mst1/2 regulation by Rassf polypeptides has been best studied in the murine T cell (Katagiri *et al*, 2006; Zhou *et al*, 2008). In resting cells, Mst1 in complex with Rassf5b/Nore1b is inactive, however, activation of chemokine or antigen receptors results in recruitment of the heterodimer to Rap1–GTP at the membrane and activation of Mst1 through a mechanism yet to be defined. Little information is available concerning regulation of other Rassf/Mst1-2 complexes and in other tissues.

Mst1 and Mst2 also become activated in cells undergoing apoptosis from a variety of stimuli. The activation mechanism is unclear however once activated, both kinases undergo cleavage by caspase3 at sites just carboxyterminal to their catalytic domains (Graves *et al*, 1998). The resultant catalytic polypeptides display an altered substrate specificity (Anand *et al*, 2008); moreover now lacking their autoinhibitory domain, the caspase-cleaved catalytic fragments are highly and constitutively active with unrestricted nuclear access (Ura *et al*, 2001).

A striking feature of Mst1/2 regulation in liver is the finding that a substantial fraction of Mst1 and some Mst2 are present as constitutively active, presumably caspase-cleaved catalytic fragments (Zhou *et al*, 2009). This contrasts with unstimulated T cells and MEFs, wherein Mst1/2 are found exclusively as the full length polypeptides, inactive unless the cells are stimulated or subjected to proapoptotic treatments. Notably however, in these cells the outputs of Mst1/2 as well as their regulation differs from that in hepatocytes. In T cells, Mst1 activation by the T-cell receptor promotes integrin clustering independently of Yap (Katagiri *et al*, 2006; Zhou *et al*, 2008). In MEFs, cell–cell contact activates Lats1/2 and promotes Yap(Ser127) phosphorylation equally well in wild-type and Mst1/2-deficient cells (Zhou *et al*, 2009). Thus the basal activity of Mst1 (especially) and Mst2 in liver seems to be uniquely high and tightly coupled to Yap inhibition, a situation that is probably of major importance to the maintenance of hepatocyte proliferative quiescence. Although further work is needed to verify the role of caspase3 in the generation of these Mst1/2 fragments, a non-apoptotic role for caspase3 in the suppression of cell proliferation and promotion of differentiation has been previously demonstrated in embryonic and hematopoietic stem cells (Fujita *et al*, 2008; Janzen *et al*, 2008) and in myoblasts (Fernando *et al*, 2002), in the latter involving Mst1 cleavage.

The mechanism underlying the activation of hepatocyte Mst1 activation before its caspase cleavage is not yet known. The finding that Alb–cre-mediated Sav1 deletion has a selective effect on oval cell proliferation points to a role for Sav1 in Mst1/2 regulation in that compartment, but implies as well that other mechanisms of Mst1/2 activation may be predominant in the hepatocytes. Although epigenetic inactivation of Rassf1A and of Rassf5b/Nore1b has been reported in HCC (Calvisi *et al*, 2006; Macheiner *et al*, 2006), whether hepatocyte Mst1/2 activation involves Rassf(1-6) polypeptides, or as yet unidentified mechanisms is not known.

## Role of Mst1/2-Yap pathway in human liver cancers

The contributions of the core Hippo pathway components to human malignancies has received limited attention thus far. Mutations in Mst1/2, Lats1/2 and Sav1 in human cancers are very infrequent in the COSMIC catalogue, however there is evidence of promoter silencing by hypermethylation in some cancer types (e.g., Lats1/2 in breast cancers; Takahashi *et al*, 2005). As to liver cancer, the most compelling information relates to Yap. Approximately 50% of human HCC's show aberrant overexpression and nuclear localisation of Yap (Dong *et al*, 2007; Zhao *et al*, 2007), a small fraction of which is attributable to *Yap* gene amplification (Zender *et al*, 2006). Cell lines derived from human CC exhibit extensive apoptosis consequent to shRNA-induced deficiency of Yap, and the murine and human liver tumours of mixed HCC/CC morphology exhibit substantially increased nuclear abundance of Yap (Zhou *et al*, unpublished observations). Thus, Yap is probably a significant oncogene is all subtypes of liver cancer.

As regards the mechanisms underlying aberrant Yap activation, our preliminary immunoblot analysis of lysates from human HCC and adjacent non-neoplastic liver (Zhou *et al*, 2009) show that ∼30% of tumours have reduced Yap(Ser127) phosphorylation, while retaining wild-type or upregulated Yap protein levels. Moreover, the cleaved, activated Mst1/Mst2 peptides are absent in a similar proportion of HCCs and the extent of pMob(Thr12) phosphorylation, a highly specific substrate of Mst1/2 (Praskova *et al*, 2008; Zhou *et al*, 2008, 2009), is greatly reduced. These findings point to a deficiency in the upstream activating input to Mst1/2 in HCC, and emphasise the importance of defining this regulation. Thus, loss of regulation upstream of Mst1/2 is a common abnormality in human HCC and may account for Yap activation in these tumours. It is not yet clear whether Yap dysregulation is a feature of the hyperproliferative states that precede HCC or is a late consequence of transformation.

## Conclusion

Mst1/2, Yap, and Sav1 are now clearly established as regulators of liver cancer pathogenesis, yet many questions remain. Of primary importance is the need to understand the mechanisms that underlie Mst1/2 activation in hepatocytes and oval cells, that is, the nature of the activating signals and the role of cell surface proteins such as the atypical cadherin Fat and sub-membrane FERM domain scaffold proteins, such as Merlin/NF2 and Expanded, elements known to be functionally associated with the *Drosophila* Hippo pathway (reviewed by Reddy and Irvine, 2008). It will be important to examine whether Mst1/2 cleavage in liver is a passive consequence of Mst1/2 activation or is itself regulated. Is Mst1/2 cleavage required for liver quiescence and does such cleavage also occur in the wild-type oval cells as well as in hepatocytes; how does cleavage influence Mst1/Mst2 subcellular localisation and target specificity. The identification of the novel Mst1/2 activated kinase that phosphorylates and inhibits Yap1 in liver is of great interest as are the mechanisms by which Sav1 controls Yap1 levels in oval cells. The contribution of Yap and other factors to oval cell overproliferation remains to be formally determined. A more comprehensive elucidation of the transcription factors and transcriptional outputs downstream of Yap in liver is needed. It is important to note that, although Yap is clearly the oncogene that is negatively regulated downstream of Mst1/2 in liver, Yap also has antiproliferative, propapototic outputs in other cellular milieus, presumably reflecting its context-dependent interactions with transcriptional regulators unrelated to those that define Yap function in the Hippo tumour suppressor pathway (e.g., p73; Matallanas *et al*, 2007; Oh and Irvine, 2010).

In terms of human liver cancers, it will be important to identify clinically useful biomarkers for deregulation of Mst1/2 signalling in human HCC and CHC. At present, Yap IHC staining of liver biopsy is commonly used (Steinhardt *et al*, 2008). Additional antibody against phospho-Mob1 used for IHC staining needs to be developed. Other important issues are to define the clinical correlates of liver cancers that show deficient Mst1/2-Yap regulation (Xu *et al*, 2009), and to determine whether there are druggable targets in this pathway, for example, upstream of Mst1/2 or downstream of Yap1. Enhancement of Mst1/2 kinase activation or inhibition of Yap expression or function may prove a valuable strategy for drug design and discovery to improve therapy for HCC patients.

## Figures and Tables

**Table 1:**
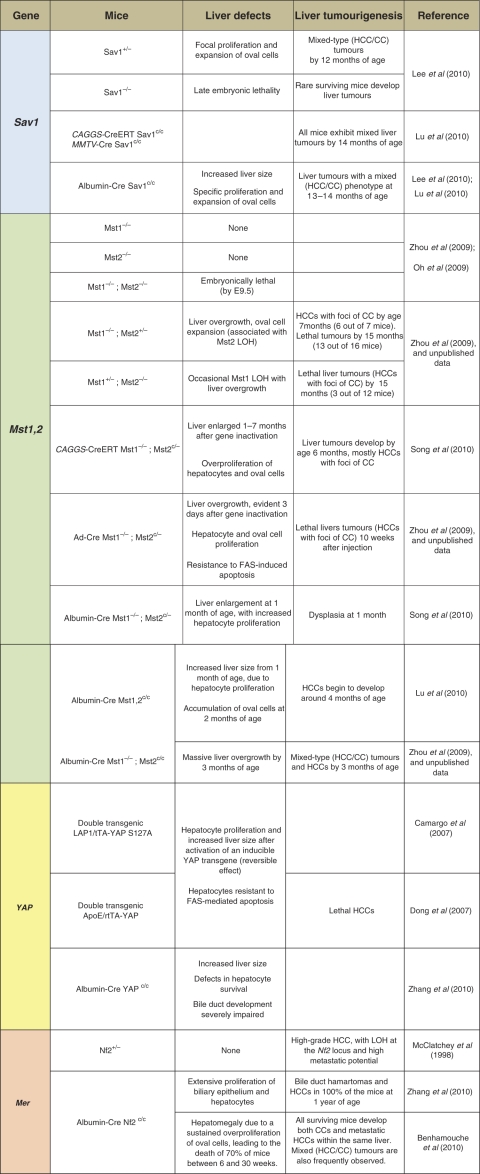
Description of the different phenotypes resulting in the inactivation of the *Sav1*, *Mst1/2*, *YAP* and *NF2/Mer* genes in mouse
